# Barriers and facilitators to colonoscopy following fecal immunochemical test screening for colorectal cancer: A key informant interview study

**DOI:** 10.1016/j.pec.2021.09.022

**Published:** 2022-06

**Authors:** Robert S. Kerrison, Elizabeth Travis, Christina Dobson, Katriina L. Whitaker, Colin J Rees, Stephen W Duffy, Christian von Wagner

**Affiliations:** a Department of Behavioural Science and Health, University College London, London, UK; b School of Health Sciences, University of Surrey, Surrey, UK; c School of Psychology, University of Leeds, Leeds, UK; d Centre for Cancer, Newcastle University, Newcastle Upon Tyne, UK; e Population Health Sciences Institute, Newcastle University Centre for Cancer, Newcastle University, Newcastle Upon Tyne, UK; f Department of Gastroenterology, South Tyneside NHS Foundation Trust, South Shields, UK; g Wolfson Institute, Queen Mary University London, London, UK

**Keywords:** Colonoscopy, Screening, Early diagnosis, Fecal immunochemical test, Qualitative research, Interviews

## Abstract

**Objectives:**

People who are referred for colonoscopy, following an abnormal colorectal cancer (CRC) screening result, are at increased risk of CRC. Despite this, many individuals decline the procedure. The aim of this study was to investigate why.

**Methods:**

As little is currently known about non-attendance at follow-up colonoscopy, and follow-up of abnormal screening results is a nurse-led process, we decided to conduct key informant interviews with Specialist Screening Practitioners ([SSPs] nurses working in the English Bowel Cancer Screening Program). Interviews were conducted online. Transcripts were assessed using inductive and deductive coding techniques.

**Results:**

21 SSPs participated in an interview. Five main types of barriers and facilitators to colonoscopy were described, namely: Sociocultural, Practical, Psychological, Health-related and COVID-related. Key psychological and sociocultural factors included: ‘*Fear of pain and discomfort associated with the procedure*’ and ‘*Lack of support from family and friends*’. Key practical, health-related and COVID-related factors included: ‘*Family and work commitments*’, ‘*Existing health conditions as competing priorities*’ and ‘*Fear of getting COVID-19 at the hospital'*.

**Conclusions:**

A range of barriers and facilitators to follow-up colonoscopy exist. Future studies conducted with patients are needed to further explore barriers to colonoscopy.

**Practice implications:**

Strategies to reduce non-attendance should adopt a multifaceted approach.

## Highlights

• A range of practical, psychological & social barriers to colonoscopy were reported.
• Psychological barriers (e.g., fear of pain) were considered to be the most pertinent.
• Several new barriers, including fear of getting and spreading COVID were described.
• The results advocate a multifaceted approach to reducing barriers to colonoscopy.
• Providing information on the risk of COVID might increase uptake during lockdowns.

## 1 Introduction

Colorectal cancer (CRC, also referred to as ‘bowel cancer’) is a leading cause of morbidity and mortality in Europe [1]. Several large randomized controlled trials (RCTs) have shown that regular fecal immunochemical test (FIT) screening, between the ages of 45 and 80, can significantly reduce the mortality of the disease among people who complete the test [2]. As a result, many European countries have implemented FIT-based screening programs for the early detection of CRC [3].

As with all screening, the extent to which the benefits of FIT screening are realized is highly dependent on the uptake of the screening test, as well as any necessary follow-up investigations (colonoscopy being the gold standard) [3]. In a recent international survey of 35 FIT screening programs, however, Selby et al. found that the mean proportion of participants with a positive FIT result who complete follow-up colonoscopy was only 79%, with completion rates ranging from 39% in the program with the lowest level of follow-up, to 100% in the country with the highest [4].

Non-attendance at colonoscopy is a major source of inefficiency within CRC screening programs and is associated with a range of adverse outcomes, including increased risk of CRC, advanced stage diagnosis and CRC death [5]. As a result, there is much current interest in understanding the reasons for lack of follow-up, and how to prevent it [6], [7].

With regards to the quantitative literature, a number of studies utilizing surveys and electronic health records have been conducted, demonstrating evidence of disparities in uptake by socioeconomic position and ethnicity [6]. While such studies are useful in terms of identifying low uptake groups, they tend to focus more on demographic, clinician, and program factors, and less on the important reasons why patients decline the test offer [6]. To this end, a wide range of qualitative studies have been performed.

To date, the majority of qualitative research examining non-attendance at colonoscopy has focussed on barriers to colonoscopy as a primary screening test for asymptomatic adults, as opposed to a diagnostic test for those with an abnormal screening result [7]. Indeed, a recent review of the literature found that, of 57 qualitative studies exploring barriers and facilitators of colonoscopy use, 54 focussed on ‘screening colonoscopy’ (only two focussed on ‘follow-up colonoscopy’), nearly all of which (n = 48) were conducted in the USA, where the delivery of screening is opportunistic, and highly different from the organized programs offered in Europe and the rest of North America [7]. Other reviews of barriers to endoscopic examinations have drawn similar conclusions. For example, in a separate review of barriers to colonoscopy, Lim et al. included studies that explored the perspectives of healthcare professionals, but were unable to find any conducted outside of North America [8]. One review (Travis et al. 9) of the barriers to flexible sigmoidoscopy screening did find several studies conducted within Europe; however, given the differences between the two tests (prep time, insertion depth, sedation options, etc.), and their indications (screening vs. follow-up), the findings are not generalizable to colonoscopy [9].

Further qualitative studies with patients and healthcare professionals are needed to better understand the barriers to colonoscopy as a follow-up test. The aim of this study, therefore, was to develop an understanding of non-attendance at follow-up colonoscopy in the context of an organized FIT-based CRC screening program, by interviewing the healthcare professionals involved in the decision-making process.

## 2 Methods

### 2.1 Participants

**Fig. 1:**
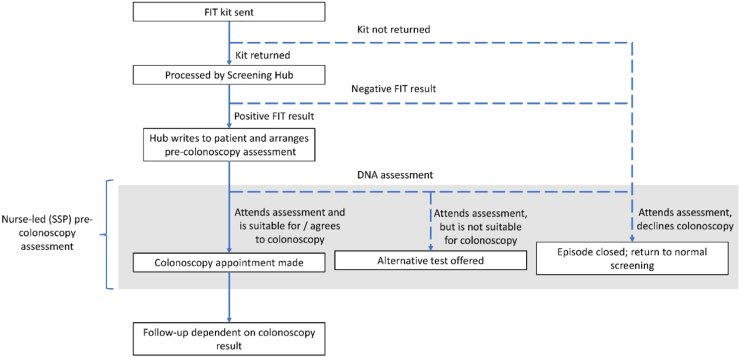
Flow chart depicting typical patient flow through the screening program (adapted from Plumb et al., 2017) and the role of the SSP.

As little is currently known about non-attendance at follow-up colonoscopy within organized FIT-based CRC screening programs, and follow-up of a positive result is a nurse-led process in the English Program, we decided to conduct key informant interviews with ‘Specialist screening practitioners’ (‘SSPs’): specialist nurses, employed by the English Bowel Cancer Screening Program (BCSP), who specialize in assessing patients’ health for follow-up colonoscopy, and provide support in deciding whether to undergo further examination (they typically assess about six patients a week - See Fig. 1 for an overview of the patient pathway and the SSPs role within it).

### 2.2 Recruitment

Participants were recruited through the SSP Knowledge Hub (using convenience sampling): an online forum accessed by approximately one third of SSPs working in England. SSPs with access to the knowledge hub were sent a participant information sheet, which outlined the purpose of the study and directed interested individuals to contact the principal investigator (RK) via email. The interviewer had no prior relationship with the participants.

### 2.3 Setting

All interviews were conducted online, between October and November 2020, using one of three virtual platforms, namely: ‘Microsoft Teams’, ‘Zoom’ and ‘NHS Attend Anywhere’.

### 2.4 Data collection

Interviews were conducted by Dr Robert Kerrison (PhD): a male Senior Research Fellow with nine years’ experience in the field of Behavioral Science and Cancer. In the first instance, 15 interviews were performed. Additional interviews were then carried out in sets of 3, until no new themes were found in the data. Interviews lasted up to 75 minutes and were conducted using a semi-structured interview guide, which was pilot tested with one SSP prior to data collection. Questions focussed on SSPs experiences dealing with FIT results, the pre-colonoscopy assessment and the colonoscopy referral (see Appendix 1). An audio recorder was used to audio record the interviews (field notes were also taken during the interviews). The recordings were anonymized, transcribed verbatim (RK), and deleted immediately after.

### 2.5 Data analysis

Two authors (RK and ET) coded a proportion (n = 4, 19%) of the transcripts, using a coding framework synthesized from the existing literature (see Appendix 2) [7]. Thematic analysis was simultaneously applied to identify new barriers and facilitators not previously described. A revised framework was subsequently developed, which led to the removal of redundant codes (i.e. codes that did not appear in the transcripts), the addition of new codes (i.e. codes that were not previously described in the coding framework) and the revision of existing codes (some codes were relabeled to better reflect the data). One author (RK) coded the remaining transcripts (n = 17, 81%) using the revised framework. Several further codes were subsequently added to the framework as new transcripts were analyzed (previously coded transcripts were then revisited to check for the presence of newly identified codes). Superordinate themes, themes and subthemes were then developed by three authors (RK + ET + CD) through an iterative process of comparing, re-examining, and grouping the codes until consensus was achieved. The superordinate themes, themes and subthemes were shared with, and considered by all authors to ensure they were consistent and apposite. The data were coded and analyzed in Excel. The number of interviews in which subthemes were identified was also reported, to help assess the extent to which they might be important. To minimize participation burden, participants were not invited to review the findings.

### 2.6 Rigor

After each stage of data analysis, two reviewers (RK and ET), plus a third reviewer (CD), discussed the thematic findings and resolved disagreements to help maintain theoretical validity (i.e., reliability of data interpretation) [10].

### 2.7 Transparency

The reporting of this interview study follows the Consolidated Criteria for Reporting Qualitative Research (COREQ) guidelines (Appendix 3) [11]. A database of the coded text is available from Open Science Framework provided for further transparency (see: https://osf.io/5fe2c/).

### 2.8 Ethics

The study was approved by University College London’s Joint Research Office (reference: 599/002) on the 6th of July 2020.

## 3 Results

### 3.1 Participant characteristics

In total, 21 SSPs participated in the study. The majority were female (n = 20, 95.2%). The mean number of years worked as an SSP was 6.6 (range: 2–13 years; Table 1). None of the participants dropped out or withdrew from the study.

**Table 1:** Sample characteristics.

**Gender**	
Female	20 (95.2)
Male	1 (4.8)
**Occupation**	
SSP	16 (76.2)
Lead SSP	5 (23.8)
**Years experience as an SSP (Mean, Range)**	
Continuous (years)	6.6 (2–13)
**Screening Center**	
St Mark’s BCSC (Northwest London)	8 (38.0)
Yorkshire and Humber BCSC	4 (19.0)
East Kent	2 (9.5)
West Kent	2 (9.5)
Blackpool	1 (4.8)
Liverpool	1 (4.8)
Leicester	1 (4.8)
Harrogate	1 (4.8)
Brighton	1 (4.8)

### 3.2 Revisions to the framework

A number of revisions to the original framework were made during the analysis process, with 39 subthemes added, 16 removed and 10 relabeled (an overview of the revisions made to the framework is provided in Table 2).

**Table 2:** Overview of original and revised conceptual frameworks (original framework synthesized from previous literature, see: Kerrison et al., 2021).

**Revised framework**	**Original framework**
**1. Sociocultural factors**	
1.1. Social support and influences	1.1. The role of positive relationships, social networks and other influences
*1.1.1. Support / lack of support from friends and family*	1.1.1. Support / lack of support from local community and social networks*
*1.1.2. Family influenced participation*	–
*1.1.3. Media coverage*	–
*1.1.4. Knowing someone with CRC*	–
*1.1.5. GP recommendation*	1.1.5. Test recommended / not recommended*
*1.1.6. Hearing other people’s experiences with colonoscopy*	–
–	*1.1.7. Patient provider relationship*
–	*1.1.8. Previous conversations with patient-provider*
1.2. Cultural and religious beliefs and attitudes	1.2. Cultural taboos and perceptions of masculinity*
1*.2.1. Unable to have a male practitioner for religious reasons*	–
*1.2.2. Colonoscopy, colon and rectum ‘culturally taboo’ topics*	1.2.2 Colonoscopy as a culturally taboo topic*
*1.2.3. Gender and engagement with healthcare*	–
*1.2.4. Fatalistic beliefs*	–
1*.2.5. Unable to accept blood products for religious reasons*	–
–	*1.2.6. Colonoscopy not ‘manly’*
–	*1.2.7. Perceived threat of bodily invasion to masculinity*
–	1.3. Past experiences and experiences of important others
–	*1.3.1 Hearing other people’s experiences with colonoscopy*
–	*1.3.2. Previous personal experiences with colonoscopy*
**2. Practical factors**	
2.1. Language barriers	–
*2.1.1. Language barriers*	–
2.2. Competing priorities and accessibility issues	2.2. Competing priorities and accessibility issues
*2.2.1. Transport / travel*	2.2.1. Difficulties getting to the appointment*
*2.2.2. Traveling / on holiday*	–
*2.2.3. Family, work and religious commitments*	2.2.2. Family and work commitment*
*2.2.4. Lack of car parking*	–
*2.2.5. Indirect costs*	–
*2.2.6. Initial invitation not received*	–
–	*2.2.7. Cost of colonoscopy*
–	*2.2.8. Colonoscopy not covered by health insurance*
–	*2.2.9. Difficulties arranging an appointment*
–	*2.2.10. Existing health conditions (Moved to ‘Health-related factors’)*
2.3. Unexpected events on the day of the appointment	–
*2.3.1. Failed bowel preparation*	–
*2.3.2. Feeling unwell*	–
*2.3.3. Personal emergency*	–
**3. Psychological factors**	
3.1. Concerns about the procedure	3.1. Concerns about the procedure
3.1.1. *Concerns about doing the bowel preparation*	*3.1.1. Concerns about doing the bowel preparation*
3.1.2. *Fear about pain and discomfort*	*3.1.2. Fear of pain and discomfort*
3.1.3. *Concerns about test invasiveness*	*3.1.3. Concerns about test invasiveness*
3.1.4. *Shame and embarrassment*	*3.1.4. Shame and embarrassment*
3.1.5. *Concerns about availability and necessity of sedation*	*3.1.5. Concerns about availability and necessity of sedation*
3.1.6. *Concerns about perforation and procedural risks*	*3.1.6. Concerns about perforation and procedural risks*
3.1.7. *Concerns about practitioner performing the test*	–
–	*3.1.8. Fear of not knowing*
–	*3.1.9. Existing health conditions interfering with ability to do the bowel preparation (Moved to ‘Health-related factors’)*
3.2. Knowledge about CRC, screening and colonoscopy	3.2. Knowledge about CRC and screening
*3.2.1. Lack of understanding that bowel cancer can be asymptomatic and the test is looking for invisible traces of blood*	3.2.1. Lack of understanding that bowel cancer can be an asymptomatic disease*
*3.2.2. Lack of awareness and understanding of colonoscopy procedure*	3.2.2. Awareness and understanding / lack of awareness and understanding of the procedure*
3.3. Emotional responses during the assessment	–
*3.3.1. Anxiety*	–
*3.3.2. Denial*	–
*3.3.3. Avoidance*	–
*3.3.4. Shock*	–
3.4. Cognitive abilities and ability to make an informed decision	–
*3.4.1. Lack of capacity*	–
*3.4.2. Low health literacy*	–
*3.4.3. Memory issues*	–
3.5. Perceived CRC risk and perceived benefits of colonoscopy	3.5. Perceived risk and perceived mortality
3.5.1. Proactive desire to stay healthy	–
3.5.2. Peace of mind	–
3.5.3. Having CRC symptoms	*3.5.3. Having CRC symptoms*
3.5.4. Having a family history of CRC	*3.5.4. Having a family history of CRC*
–	*3.5.5. Cancer fear*
–	*3.5.6. Perceived mortality and potential to benefit from colonoscopy*
–	3.6. Enhanced peace of mind
–	*3.6.1. Colonoscopy provides long lasting peace of mind*
–	*3.6.2. Colonoscopy examines whole bowel*
–	3.7. Enhanced peace of mind
–	*3.7.1. Lack of interest and procrastination*
–	3.7.2. Proactive desire to stay healthy*
–	3.8. Post hoc rationalization for abnormal screening result
–	*3.8.1. Providing an alternative explanation for the test results*
–	*3.8.2. Distrust in the screening result*
**4. Health related factors**	
4.1. Existing health conditions and medical history affecting clinical eligibility to have the test	–
*4.1.1. Clinically ineligible or inappropriate*	–
4.2. Existing health conditions and medical history affecting patient willingness to have the test	–
*4.2.1. Recent Colonoscopy*	–
*4.2.2. Existing health condition interfering with ability to do the bowel preparation*	–
*4.2.3. Previous personal experiences with colonoscopy and other medical investigations*	–
*4.2.4. Existing health conditions as a competing priority*	–
**5. COVID-Related factors**	
5.1. Impact of COVID	–
*5.1.1. Fear of getting COVID*	–
5*.1.2. Unable to leave the house due to shielding*	–
5*.1.3. Fear of spreading COVID*	–
5.2. Impact of COVID measures	–
*5.2.1. Unable to get in contact with patients*	–
*5.2.2. Patients unable to bring friend / family for emotional support*	–
*5.2.3. Patient and household required to self-isolate prior to procedure*	–

* Indicates that the theme or subtheme has been relabeled in the revised model.

### 3.3 Description of themes

In total, five main types of barriers and facilitators were identified: Sociocultural, Practical, Psychological, Health-related and COVID-related. Psychological and sociocultural factors centered on intrinsic constructs, such as cultural taboos, concerns about the procedure, and knowledge about CRC. Conversely, practical, health-related and COVID-related factors centered on more extrinsic constructs, such as indirect costs associated with attending the pre-colonoscopy assessment or colonoscopy appointment, existing health conditions, and shielding.

**Fig. 2:**
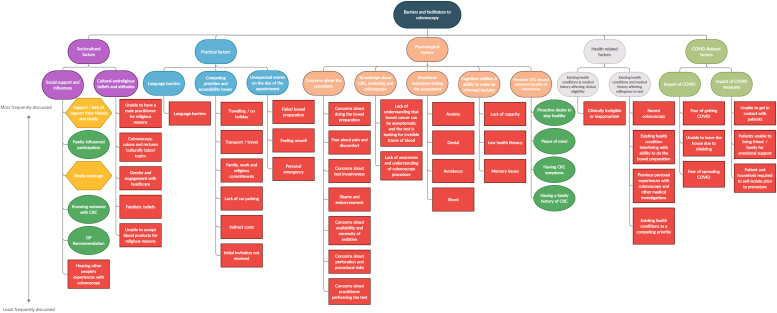
Tree diagram of the revised coding framework, showing the relationships between themes, higher order themes and superordinate themes. Themes in red (squares) represent barriers of colonoscopy, themes in yellow (hexagons) represent themes which could be either barriers or facilitators of colonoscopy, and themes in green (ovals) represent facilitators of colonoscopy. Themes that were identified most frequently are listed first within their respective groups. For interpretation of the references to color in this figure legend, the reader is referred to the web version of this article.

Fig. 2 provides a diagrammatic overview of the barriers and facilitators of colonoscopy use. The following provides a detailed description of the barriers and facilitators identified; illustrative quotes and the number of SSPs who discussed each of the barriers and facilitators are presented in Table 3.

**Table 3:** Example quotes.

**Subtheme** (number of participants)	**Example quotations**
**1. Sociocultural factors**	
1.1. Social support and influences	
*1.1.1. Support / lack of support from friends and family (21)*	*“”"If they're very anxious, and they, they can't take it in, there's somebody else there to actually take that in, and also, having somebody there as well will often help to resolve transport issues as well, because they'll say, "Oh, no, I'll take you" or "we know ‘so and so’ will take you"* (Participant 12)
*1.1.2. Family influenced participation (10)*	“*I find the men who have the wives, the wives will say "he's having it, and that's it", you know, they've not choice.*” (Participant 1)
*1.1.3. Media coverage (9)*	“*We do see an upsurge when celebrities say that they've been diagnosed with bowel cancer. You know, all of a sudden, it's like, "oh, it is real". You know?*” (Participant 11)
*1.1.4. Knowing someone with CRC (4)*	*I think a lot of patients say that they've had a family member or a friend, a close friend, that's had bowel cancer, that's triggered it off for them to do the test kit*” (Participant 14)
*1.1.5. GP recommendation (4)*	“*And then what we'll do is we'll kind of reiterate it with all the pros and cons of having the colonoscopy, explain the colonoscopy to the patient. And then they'll say, "Oh, I want to go back and I want to discuss it with my GP", particularly if they're a very anxious sort of person*” (Participant 17)
*1.1.6. Hearing other people’s experiences with colonoscopy (3)*	“*They may say, "Oh, well, my dad had this procedure and said it was so painful". Then they're worried as well about painfulness of colonoscopy*” (Participant 1)
1.2. Cultural and religious beliefs and attitudes	
1*.2.1. Unable to have a male practitioner for religious reasons (18)*	*Culturally, they're not supposed to be seen or touched by anybody other than their husband*” (Participant 17)
*1.2.2. Colonoscopy, colon and rectum ‘culturally taboo’ topics (6)*	“*I have found Muslims to be very, very hard in accepting colonoscopy, or any investigation, especially related to the bowels. I have no idea. I do try to explore with patients, but I generally get shut down when patients don't want to proceed. I know straightaway, no matter of talking will help. So these two populations, the black population and Muslims are very, very hard to work around*” (Participant 8)
*1.2.3. Gender and engagement with healthcare (5)*	“*Um, you tend to find, like I said, the gentleman tend to be a little more anxy than the ladies I find, really. That's my experience. Um, men don't go to the doctor, you know, I don't know how many times I've heard them say that. "Oh, yeah love I'm fit as a fiddle love. I haven't been to doctors in 30 years, and duh dee duh*.” (Participant 14)
*1.2.4. Fatalistic beliefs (2)*	“*The only thing I can think of, but we haven't got a huge population of, and they tend not to be in our age bracket, is the Gypsy traveler community, which we've got here. But they tend to be younger population, the life expectancy isn't as high as, but it's. if the bowel screening age came down, then yes, it would. But there is that, if it's, you leave well alone, you don't mess about. So whatever, it's God's, God's way. Whatever will be will be*” (Participant 9)
1*.2.5. Unable to accept blood products for religious reasons (1)*	“*99% of the time, we will do the consent there [during the pre-colonoscopy assessment], and the patient will sign it, unless they're a Jehovah Witness”* (Participant 1)
**2. Practical factors**	
2.1. Language barriers	
*2.1.1. Language barriers (21)*	“*Normally we find someone, but the most difficult language is Nepalese. Even in language line, sometimes we can't find Nepalese interpreter at that time.*” (Participant 6)
2.2. Competing priorities and accessibility issues	
*2.2.1. Transport / travel (21)*	*“People from outside of Ashford / Romney Marsh area will not travel to Margate. It's a good hour and 20 min’ drive*” (Participant 19)
*2.2.2. Traveling / on holiday (12)*	“*Some people don't attend first one and then they may come to the second one. And I usually ask them, I say, ”Oh, you never came last time was everything, okay?” And it could be that they're on holiday, because they get the appointment quite quick. And they may actually be on holiday, and the appointment is posted to them and they have no idea they've got the appointment*” (Participant 1)
*2.2.3. Family, work and religious commitments (11)*	“*People have a life, they have commitments, they have obligations. People have to work. If like, I am told I need to have a colonoscopy, I cannot take two days off work. I've got young children. So you have. they have these concerns about how are they going to adjust the life around it*” (Participant 8)
*2.2.4. Lack of car parking (10)*	“*Um, handicapped parking, the blue zone parking, is not good*” (Participant 19)
*2.2.5. Indirect costs (8)*	“*Leeds patients, quite often, are more likely to want to stay within the Leeds city hospitals. A lot of them might not have access to a car, they can't afford taxis*” (Participant 20)
*2.2.6. Initial invitation not received (4)*	“*Very often they've been away, they didn't get the letter in time. They've been staying at a family members. They've moved house and not let the GP know. Um, you know.*” (Participant 9)
2.3. Unexpected events on the day of the appointment	
*2.3.1. Failed bowel preparation (8)*	“*Um, some of the reasons might be with the prep, that they haven't finished it, or they started it, and, you know. They start it and they, they like, they've eaten, they haven't read properly the instructions and they've eaten and. maybe they come for the appointment, but the colonoscopy doesn't happen, because they haven't followed properly the instructions*” (Participant 5)
*2.3.2. Feeling unwell (6)*	"*Um, some people have cancelled last minute. And they're usually the ones... it's because of anxiety, or some of them are genuinely sick, they're actually just not well, and usually they're the ones who cancel colonoscopy.*" (Participant 1)
*2.3.3. Personal emergency (3)*	“*They need to attend a funeral*” (Participant 1)
**3. Psychological factors**	
3.1. Concerns about the procedure	
*3.1.1. Concerns about doing the bowel preparation (19)*	“*Um, some of them don't like taking the laxatives, if they have previous experience. So they say, "Oh, I hate having bowel prep, procedure itself is okay*"” (Participant 6)
*3.1.2. Fear about pain and discomfort (18)*	“*Frequently asked will be:* “*Is it painful?, with the procedure. That's always the frequently asked question. “Will the procedure be painful?” Yeah*” (Participant 2)
*3.1.3. Concerns about test invasiveness (10)*	“*Yes, there are a few of them who wants CT colonography. They will say yeah, because it's less invasive and all that*” (Participant 3)
*3.1.4. Shame and embarrassment (9)*	“*I think it's embarrassment of coming to the endoscopy unit and being on a trolley with other people around with your bottom on show. It's not quite the same as having a cardiac stent and heart surgery and things. It doesn't quite go into the same category*” (Participant 13)
*3.1.5. Concerns about availability and necessity of sedation (9)*	“*Some patients think sedation means general anesthetic. We think. there is a fear about general anesthetic. It is. is “once I go to sleep, I'll never wake up””* (Participant 8)
*3.1.6. Concerns about perforation and procedural risks (5)*	“*Once they consent, we, you know, try and reassure them and say this is worst case scenario, it's highly unlikely, but they do get worried when they hear about the risk of bleeding and perforation. That does make them worried*” (Participant 1)
*3.1.7. Concerns about practitioner performing the test (5)*	“*I did have few questions like "who's going to do my procedure? I don't want a junior… I don't want anyone to practising on me" and things like that*” (Participant 3)
3.2. Knowledge about CRC, screening and colonoscopy	
*3.2.1. Lack of understanding that bowel cancer can be asymptomatic and the test is looking for invisible traces of blood (16)*	“*Some patients say there's “nothing wrong with me. If I get anything. if anything's wrong, then I'll go and see my doctor”. But we say “sometimes polyps or bowel cancer doesn't give you any symptoms”. We do explain all that. That's why we do the test kit because it picks up the blood from them. But they still say “well, I've not got anything wrong with me”* (Participant 13)
*3.2.2. Lack of awareness and understanding of colonoscopy procedure (7)*	“*We get a lot of autistic patients. Um, sometimes. And they have different needs, and you just have to tailor whatever you have with that. And they might want to see the room before they come in, decide whether they want to have it done. And they might want to see the actual scope to see what it looks like because their interpretation quite often is "you're going to put a camera on my bottom, but is it going to be a box brownie?*"” (Participant 17)
3.3. Emotional responses during the assessment	
*3.3.1. Anxiety (20)*	“*I would say a good 90% are anxious. Because you've told them that. I mean, they've done this kit thinking it's gonna come back normal. And it comes back and says there's blood in it could be bowel cancer. So before they come into the clinic, they're thinking, "Oh, my God, they've told me I've got bowel cancer"* (Participant 12)
*3.3.2. Denial (19)*	“*They ask for another test, because that day, “I had to eat whatever”. And so… And also, “I was very constipated, and I want to do another test a different day, because I'm sure it won't come back positive””* (Participant 5)
*3.3.3. Avoidance (6)*	“*I just said, "so how do you feel about colonoscopy?" And he said, "Nay lass, why would I want to do that?*" (Participant 12)
*3.3.4. Shock (2)*	“*I think it goes back to they've done the test and not expected to get a positive result. They thought it would be a negative result, the minute it becomes positive "Oh, I can't do that. I can't do that*"” (Participant 16)
3.4. Cognitive abilities and ability to make an informed decision	
*3.4.1. Lack of capacity (17)*	“*The other problem that we have is when people don't, maybe don't have capacity, and carers and things think they're doing the right thing and do the kits for them. And then they're positive and then, you know, again, it's not appropriate screening isn't always appropriate for those people*.” (Participant 10)
*3.4.2. Low health literacy (8)*	“*Some of the more rural and affluent read more and are more informed before they come. Some of the. some of the. because of the reading age in Hull is about seven, eight. A lot of people in the inner cities might struggle with the booklets that go through. I mean, we sometimes have to use easy read*.” (Participant 12)
*3.4.3. Memory issues (8)*	“*They forget things, but then we've got the medical notes, we get GP summary, so we get a summary of all their care, their history, but it's, it can be challenging, if they don't have all the information in the clinic, because then you may not be able to book the procedure until you get all the medical information.*” (Participant 1)
3.5. Perceived CRC risk and perceived benefits of colonoscopy	
*3.5.1. Proactive desire to stay healthy (13)*	“*I think they make a conscious decision to come in and have the procedure done, because they think of it as part of keeping themselves well*” (Participant 18)
*3.5.2. Peace of mind (12)*	“*Number one reason, I think is that they want to know if they've got bowel cancer, or if it's all clear. That's why I think a lot of them would agree to a date*” (Participant 4)
*3.5.3. Having CRC symptoms (10)*	“*Some of them may have, you know, had problems with their bowel, even though, you know, we're screening. So they'll be like, "Oh, I know, I need to have this done", you know?*” (Participant 13)
*3.5.4. Having a family history of CRC (7)*	*"Family History. Um, doesn't necessarily have to be colon or rectal cancer. If they've got somebody in their family that they're close to that has cancer, or has had cancer or has died from cancer, they're more likely to have an investigation done because they've, they've got that knowledge that it's a good thing to get these things looked at and sorted out sooner rather than later."* (Participant 18)
**4. Health related factors**	
4.1. Existing health conditions and medical history affecting clinical eligibility to have the test	
*4.1.1. Clinically ineligible or inappropriate (16)*	“*For example, patients who had a very recent heart attack. They. so. they were on the blood thinners for less than a year. It's unsafe to stop it.* ” (Participant 4)
4.2. Existing health conditions and medical history affecting patient willingness to have the test	
*4.2.1. Recent Colonoscopy (12)*	“*Some of them, like I said, had colonoscopy last month, or something. So we… essentially, they would refuse it, they don't they don't want a similar test that soon*” (Participant 4).
*4.2.2. Existing health condition interfering with ability to do the bowel preparation (10)*	“*If they've got severe mobility problems, or really bad COPD, and getting up and down to the toilet all the time is going to cause them lots of issues, then […] they're not impressed with it*” (Participant 20)
*4.2.3. Previous personal experiences with colonoscopy and other medical investigations (9)*	“*Some people have had bad experiences at other places. So they may have had colonoscopies somewhere else, and they've had a bad experience, they found it really painful, and because of that bad experience, […] they kind of then think this is going to go wrong*” (Participant 1)
*4.2.4. Existing health conditions as a competing priority (6)*	“*He's got other health problems that are more important that he wants sorted out before he comes in for a colonoscopy. So he hasn't had his colonoscopy yet*” (Participant 18)
**5. COVID-Related factors**	
5.1. Impact of COVID	
*5.1.1. Fear of getting COVID (13)*	*“They're more worried about COVID than getting a cancer*” (Participant 7)
5*.1.2. Unable to leave the house due to shielding (2)*	“*There were a few that said that they were shielding and they didn't want to come because they were shielding*” (Participant 9)
5*.1.3. Fear of spreading COVID (1)*	“*They're too afraid to expose to the hospital, because one of their family members maybe is very ill*” (Participant 7)
5.2. Impact of COVID measures	
*5.2.1. Unable to get in contact with patients (4)*	“*A lot of them, because our number comes up ‘private number’, they won't answer*” (Participant 1)
*5.2.2. Patients unable to bring friend / family for emotional support (3)*	“*Um, what else we do let patients to bring a relative, although with COVID, we don't allow relatives anymore*” (Participant 5)
*5.2.3. Patient and household required to self-isolate prior to procedure (2)*	“*Um, I have had a patient more recently, um, who was due to have a colonoscopy, but they broke the isolation rules at that point for the place where they were having the colonoscopy, so we had to cancel it*” (Participant 20)

#### 3.3.1 Sociocultural factors

##### 3.3.1.1 Social support and influences

SSPs reported *Support from friends and family* to be central to many people’s decisions, not only about follow-up colonoscopy, but also in relation to their decision about attending the pre-colonoscopy assessment, for which they often rely on friends and family for transport to the clinic, or as emotional support. In some instances, family members were reported to go beyond the provision of practical and emotional support, and actively influenced the individual’s decision making, with *Family influenced participation* reported by a number of SSPs.

For some patients, the primary care provider was said to play an important role in the decision to attend colonoscopy, specifically with regards to receiving a *GP Recommendation*. SSPs also reported that hearing stories in the media, or from other people, has a significant effect on patients’ willingness to attend colonoscopy. SSPs frequently made reference to *Media Coverage* of celebrity stories as having a positive influence; conversely, *Hearing other people’s experiences with colonoscopy* was described as a barrier to follow-up colonoscopy, with (negative) experiences of family and friends often deterring individuals from having the test.

*Knowing someone with CRC* was also described as an important motivating factor, particularly where the person affected is a close friend or family member.

##### 3.3.1.2 Cultural and religious beliefs and attitudes

SSPs described a range of cultural barriers to colonoscopy. Foremost among these was the fact that *Colonoscopy, colon and rectum are ‘culturally taboo’*, particularly among religious and ethnic minority groups, including Muslim and Black patients.

*Fatalistic beliefs* were a less frequently reported cultural barrier to colonoscopy, but one which was considered particularly salient to the White Gypsy / Irish Traveler community. SSPs also reported that men are generally more reluctant to engage with colonoscopy, but that this cultural phenomenon was not exclusive to colonoscopy, but other forms of healthcare as well (*Gender and engagement with healthcare*).

In addition to cultural barriers to colonoscopy use, SSPs described several religious barriers. Foremost among these was being *Unable to have a male endoscopist*, which was a barrier that was specifically reported by Muslim women, who did not want to be seen or touched by a male other than their husbands, meaning that they requested that only female staff, including interpreters, be present during the procedure, and sometimes the pre-colonoscopy assessment as well. Jehovah’s Witnesses were reported to face the additional barrier to colonoscopy of being *Unable to accept blood products*, which meant that some would choose not to undergo the colonoscopy in case this was required.

#### 3.3.2 Practical factors

##### 3.3.2.1 Language barriers

Language was described to be a key barrier for patients whose first language was not English and was also reported as a barrier for patients who had hearing difficulties or previously experienced a stroke. This barrier manifested itself in a number of ways, from patients missing the pre-colonoscopy appointment as the family / friend who usually interprets their mail for them was away at the time of invitation, to SSPs being unable to conduct the pre-colonoscopy assessment as no interpreter had been organized (some centers require an NHS interpreter to be translate the assessment if the patient does not speak English).

##### 3.3.2.2 Competing priorities and accessibility issues

SSPs discussed how competing priorities, such as *Family, work and religious commitments*, could act as barriers to colonoscopy. Ramadan was reported to be a difficult time for Muslims to attend colonoscopy, as the required fasting makes it difficult to complete bowel preparation. Some participants also miss the pre-colonoscopy clinic as they are *Traveling / on holiday* at the time, although SSPs reported that these patients usually rebook.

The location of the hospital/clinic was reported to be a barrier to colonoscopy for a number of reasons*. Transport/travel* was often a barrier to attendance because of the time and/or distance patients have to travel to attend, along with the *Indirect costs* associated with traveling, and the problem of a *Lack of car* parking and the expense of on-site parking facilities.

SSPs also reported that patients sometimes did not attend the initial pre-colonoscopy appointment because the *Initial invitation was not received;* however, these individuals usually received the second invite, and attended that appointment, so this only incurred a short delay (see Fig. 1 for an overview of the invitation pathway).

##### 3.3.2.3 Unexpected events on the day of the appointment

SSPs reported a range of events that can occur on the day of the appointment and sometimes act as barriers to colonoscopy. Such events include *Feeling unwell*, *Personal emergency* and *Failed bowel preparation.* With the exception of failed preparation, all were sufficient to prevent the individual from attending their colonoscopy appointment, although SSPs indicated that most would call and rebook. For those individuals with failed preparation, however, it was not until they got to the hospital and attended their appointment that it became apparent the bowel prep had failed. In most of these cases, SSPs reported the bowel prep failed because the patient had only partially followed the instructions Fig. 2.

#### 3.3.3 Psychological factors

##### 3.3.3.1 Concerns about the procedure

SSPs reported a wide range of patient concerns about the procedure during the pre-colonoscopy assessment, some of which related to experiential aspects of the procedure, while others related to possible treatments and outcomes. Among the most frequently reported were *Concerns about the bowel preparation*, including concerns about incontinence on the journey to the hospital, having to fast in order to do the bowel preparation, and the volume and taste of bowel preparation. The bowel preparation was said to be a particular concern for people who had previously had a colonoscopy. Indeed, SSPs commented that those patients often found the bowel prep to be worse than the colonoscopy itself.

*Fear of pain and discomfort* was another prominent barrier to colonoscopy use. When asked whether there were any questions patients frequently ask during the assessment, SSPs first response consistently was that patients ask if it will be painful. *Concerns about the availability and necessity of sedation* were also common, with patients wanting to know whether they can be ‘knocked out’ during the procedure, and in some instances, were worried that they might not wake up again.

*Concerns about test invasiveness* were frequently cited as a key barrier to colonoscopy, with concerned patients often requesting a scan instead. *Concerns about perforation and procedural risks* were also reported. When discussing the risks of the procedure, SSPs reported that patients often presented *Concerns about the practitioner performing the test,* stressing that they ‘did not want anyone practicing on them’.

*Shame and embarrassment* were commonly reported by SSPs as being patient barriers to follow-up colonoscopy, with patients being embarrassed about ‘having an accident’ or ‘being exposed’, during the colonoscopy, as well as having to discuss 'bowels and bowel habits', during the assessment.

##### 3.3.3.2 Knowledge about CRC, screening and colonoscopy

Several SSPs reported that patients often state that they do not feel as though they need colonoscopy, as they ‘don’t have any symptoms’ and ‘can’t see any blood when checking their stool’ (*Lack of understanding that bowel cancer can be asymptomatic and the test is looking for invisible traces of blood*). SSPs also reported that a lack of knowledge about the procedure often proved problematic, with patients having no concept when being told that a camera would be used to visualize the large bowel (*Lack of awareness and understanding of colonoscopy procedure*).

##### 3.3.3.3 Emotional responses during the assessment

SSPs reported that patients exhibited a wide range of emotions during the assessment, several of which acted as barriers to follow-up colonoscopy. *Anxiety* was commonly reported by SSPs, who discussed how the overwhelming majority of patients are anxious, with many believing that they have cancer.

Another emotional response that SSPs frequently reported patients exhibiting was *Denial*, as a number of patients do not believe the result of their screening test, often providing alternative explanations as to why it was positive (e.g., hemorrhoids). In those instances, SSPs reported that patients did not want to proceed with colonoscopy, and sometimes wanted to repeat the screening test instead. SSPs also reported that a number of patients experienced *Shock*, as they said patients often completed the screening test expecting the result to be negative, and so were not prepared for a positive result. *Avoidance* was also reported as an emotional barrier to colonoscopy, with SSPs highlighting how some patients ‘would rather not know’ and ‘simply did not want to have colonoscopy’.

In all instances, SSPs reported ‘having to work hard’ to reassure patients and help them overcome their emotions, in order to deliver an effective assessment.

##### 3.3.3.4 Cognitive abilities and ability to make an informed decision

SSPs described several cognitive abilities that were required to make an informed decision to undergo colonoscopy. *Lack of capacity* (for instance, in relation to dementia, Alzheimer’s, or patients with learning difficulties), *Low Health Literacy* and *Memory issues* were all said to present SSPs with significant challenges in proceeding with colonoscopy. With regards to lack of capacity and memory issues, SSPs said it was often necessary to hold a ‘best interest’ meeting, where the individual carer, along with a panel of experts, including an SSP, would make a decision on behalf of the patient. Health literacy issues were different in that patients could make a decision for themselves, but often needed the information presented to them differently (e.g., via ‘easy read’ materials).

##### 3.3.3.5 Perceived CRC risk and perceived benefits of colonoscopy

Elevated risk perception was a key motivating factor for many patients, with these patients more likely to book and attend colonoscopy. Several SSPs indicated that *Having a family history of CRC* or *Having CRC symptoms* were frequently stated as reasons for accepting and attending colonoscopy. Perceived benefits of having the colonoscopy procedure were also reported to be key motivating factors for accepting and attending the colonoscopy procedure. SSPs indicated some patients feel that the test will bring *Peace of mind* and for others fed into their behaviors to maintain good health through their *Proactive desire to stay healthy.*

#### 3.3.4 Health-related factors

##### 3.3.4.1 Existing health conditions and medical history affecting clinical eligibility to have the test

SSPs explained that some patients are *Clinically ineligible or inappropriate,* either because they have an existing health condition, or they are on certain medication, such as blood thinners. In these instances, SSPs described having to discuss how best to proceed with a consultant. Responses varied, with some patients being recommended no follow-up, and others CT colonography.

##### 3.3.4.2 Existing health conditions and medical history affecting patient willingness to have the test

Even among patients who are clinically eligible, existing health conditions and the individual’s medical history were still reported to play an important role in a patient’s decision to proceed to colonoscopy. SSPs discussed how, for some patients, *Existing health conditions as a competing priority* prevented progression to colonoscopy, whereas the perceived impact of an *Existing health condition interfering with ability to do the bowel preparation* served as a significant barrier for others.

Finally, patients who had previous colonoscopies often declined follow-up colonoscopy through the BCSP, either because they felt it was unnecessary because they’d had a *Recent colonoscopy,* or because they’d had negative *Previous personal experiences with colonoscopy or other medical investigations*.

#### 3.3.5 COVID-related factors

##### 3.3.5.1 Impact of COVID

SSPs reported several COVID-related barriers to colonoscopy, including being *Unable to leave the house due to shielding* and *Fear of getting COVID*. In some instances, the ‘fear of getting COVID’ was reported to be greater than the fear of a possible colorectal cancer diagnosis. COVID-related fear sometimes extended beyond the individual patient, to include *Fear of spreading COVID,* whereby individual’s (and their households) were concerned about spreading COVID to their family and friends, (in some cases there was pressure from family and friends for them not to attend).

##### 3.3.5.2 Impact of COVID measures

In addition to COVID itself presenting barriers to colonoscopy, the measures implemented to mitigate the risk of COVID were also reported to present barriers to colonoscopy. For example, several centers reported that delivering pre-colonoscopy assessments over the phone (i.e. to reduce the risk of COVID) led to a lower uptake, primarily because the caller ID shows the SSPs as calling from a private number, from which many people were thought to be reluctant to take calls (SSPs reported they had more success when they called from a non-hospital phone). Having to self-isolate prior to the colonoscopy also led to issues, with patients’ appointments frequently needing to be rescheduled, as individuals, or members of their household, had not shielded during the required period.

## 4 Discussion and conclusion

### 4.1 Discussion

#### 4.1.1 Main findings

This study identified five main types of barriers and facilitators of colonoscopy use: sociocultural, psychological, practical, health-related and COVID-related. Of these, Psychological factors appeared to be the most important barriers. Specifically, ‘concerns about the procedure’ were identified the most frequently (‘concerns about doing the bowel preparation’ and ‘fear about pain and discomfort’ in particular), followed by emotional responses during the assessment (e.g., ‘Anxiety’ and ‘Denial’) and ‘Cognitive abilities and ability to make an informed decision’ (e.g., ‘Capacity’). Psychological factors also appeared to be the most important facilitators of colonoscopy use, with ‘perceived risk and perceived mortality’ being the most frequently discussed.

Importantly, this study identified barriers and facilitators that were specific to colonoscopy among people who receive a positive FIT result. Some of the identified barriers were applicable to a broad range of patients, such as ‘lack of support from friends and family’ and ‘emotional states’, while others, such as those relating to modesty and the role of male staff in Muslim women’s decision-making, were specific to certain patient groups.

#### 4.1.2 Comparison with existing literature

The results of this study add to the findings of our previous review [7] in several ways. First, by capitalizing on the experience of SSPs, the present study identifies unique barriers for specific patient groups, not previously interviewed, including Jehovah’s Witnesses and Muslims (e.g., being unable to accept blood products). Second, it identifies unique contextual issues, not pertinent to screening colonoscopy, such as emotional reactions to receiving an abnormal FIT result (e.g., Shock that the test result was positive, Denial that the test result was accurate). Third, it identifies cultural barriers, within the UK, relating to gender and engagement with healthcare (with men living in the UK being less likely to engage in healthcare than their female counterparts) [12].

This review also identifies a number of COVD-related barriers to colonoscopy, including ‘fear of getting COVID at the hospital’ and ‘fear of spreading COVID to others’. The findings are consistent with Rees et als'. hypothesis, that: “*Anxiety about COVID-19, family pressures, logistical considerations, such as carer responsibilities, and travel to and from the hospital while adhering to social distancing, might also be barriers*” [13]. Providing patients with information regarding the risk of contracting COVID (~1 in 200) might reassure patients about the risks of getting and spreading COVID [14], [15], thereby facilitating uptake during possible future lockdowns. Further research is needed to test this hypothesis.

Finally, it is important to note that, while this study identified several unique barriers to follow-up colonoscopy (e.g., ‘Denial about the FIT result’, ‘Lack of understanding that bowel cancer can be an asymptomatic disease and the test is looking for invisible traces of blood’, etc.), it also identified a number of barriers common to primary colonoscopy and sigmoidoscopy screening. For example, in a recent review of the literature, Lim et al. found that a lack of ‘Social support’, ‘Knowledge’ and ‘Perceived risk’ were all barriers to primary colonoscopy use [8]. Similarly, Travis et al. found ‘shame and embarrassment’, ‘procedural pain and discomfort’ and ‘competing priorities’ all inhibited primary sigmoidoscopy use in a review of the barriers to sigmoidoscopy screening [9]. Given the similarities between primary endoscopy and follow-up, it’s possible that some interventions, designed to address barriers for one indication, could address barriers for another. Consideration should be given to this hypothesis when testing interventions to promote colonoscopy use.

#### 4.1.3 Implications for future research

This study has several additional implications for future research. First, further qualitative research with patients and members of the public is needed to verify the results of this study and to explore the issues from the perspectives of service users. Second, quantitative research is needed to understand how barriers and facilitators interact with one another, and which of the perceived barriers and facilitators are significantly associated with non-attendance at colonoscopy. Third, randomized controlled trials of complex interventions, which target a range of practical, psychological and sociocultural barriers are required to identify effective strategies to reduce barriers and improve colonoscopy attendance. Adopting a theoretical framework would be particularly useful in relation to this last item. For example, the Theoretical Domains Framework allows researchers to map psychological targets onto a framework that, in turn, could be used to identify behavior change techniques that are potentially effective at modifying those targets [16]. Previous research based on this approach has been effective at changing a range of behaviors, and is proposed to offer the best approach to changing health behaviors [16], [17], [18].

#### 4.1.4 Limitations

This study has several limitations. Most importantly, it was conducted with SSPs, as opposed to patients. As such, the findings may not reflect the full range of barriers and facilitators perceived by patients, only those patients choose to disclose to SSPs. Furthermore, SSPs were recruited from the SSP Knowledge Hub. As such, the possibility of there being some selection bias cannot be dismissed. Finally, SSPs from only a proportion of screening centers participated in the study. As such, the findings may not reflect additional barriers and facilitators specific to external contexts and patient groups.

#### 4.1.5 Strengths

This study also has a number of strengths. Most importantly, following each stage of data analysis, two reviewers (RK and ET), plus a third reviewer (CD), discussed the thematic findings and resolved disagreements through discussion to help maintain theoretical validity (reliability of data interpretation) [19]. Moreover, we used framework analysis to inform our interpretation of the data. This method of analysis is not aligned with a particular epistemological, philosophical, or theoretical approach, and can be adapted for use with many qualitative approaches that aim to generate themes without bias [20]. Finally, pragmatic validity (efficacy and transferability of findings) was improved by inclusion of participant characteristic tables, providing context around the individuals, allowing readers to judge the usefulness of the findings [21].

### 4.2 Conclusion

The results imply that a range of barriers to follow-up colonoscopy exist, with psychological barriers being the most pertinent among these. Future studies, conducted with patients and members of the public, are needed to explore the barriers to colonoscopy further.

### 4.3 Practice implications

Complex interventions, which address a range of psychological, practical and sociocultural barriers to follow-up colonoscopy are required to reduce non-attendance and improve service delivery.

## CRediT authorship contribution statement

**Robert Kerrison:** Conceptualization, Methodology, Formal analysis, Investigation, Data curation, Writing – original draft, Writing – review & editing, Project administration, Funding acquisition. **Elizabeth Travis:** Methodology, Formal analysis, Writing – original draft. **Christina Dobson:** Methodology, Formal analysis, Writing – original draft. **Katriina Whitaker:** Conceptualization, Methodology, Writing – review & editing, Funding acquisition. **Colin Rees:** Conceptualization, Methodology, Writing – review & editing, Funding acquisition. **Stephen Duffy:** Conceptualization, Methodology, Writing – review & editing, Funding acquisition.**Christian von Wagner:** Conceptualization, Methodology, Writing – review & editing, Supervision, Funding acquisition.
