# Supplementary material for: Barriers and facilitators to colonoscopy following fecal immunochemical test screening for colorectal cancer: A key informant interview study
Source: Patient Educ Couns. 2022 Jun;105(6):1652–62. doi: 10.1016/j.pec.2021.09.022 (PMC9214549; doi:10.1016/j.pec.2021.09.022)

Figure 2. Tree diagram of the initial coding framework, showing the relationships between themes, higher order themes and superordinate themes. Themes in red (squares) represent barriers of colonoscopy, themes in yellow (hexagons) represent themes which could be either barriers or facilitators of colonoscopy, and themes in green (ovals) represent facilitators of colonoscopy. Themes that were identified most frequently are listed first within their respective groups. Themes highlighted in grey are specific to a particular context, indication or demographic (labelled accordingly

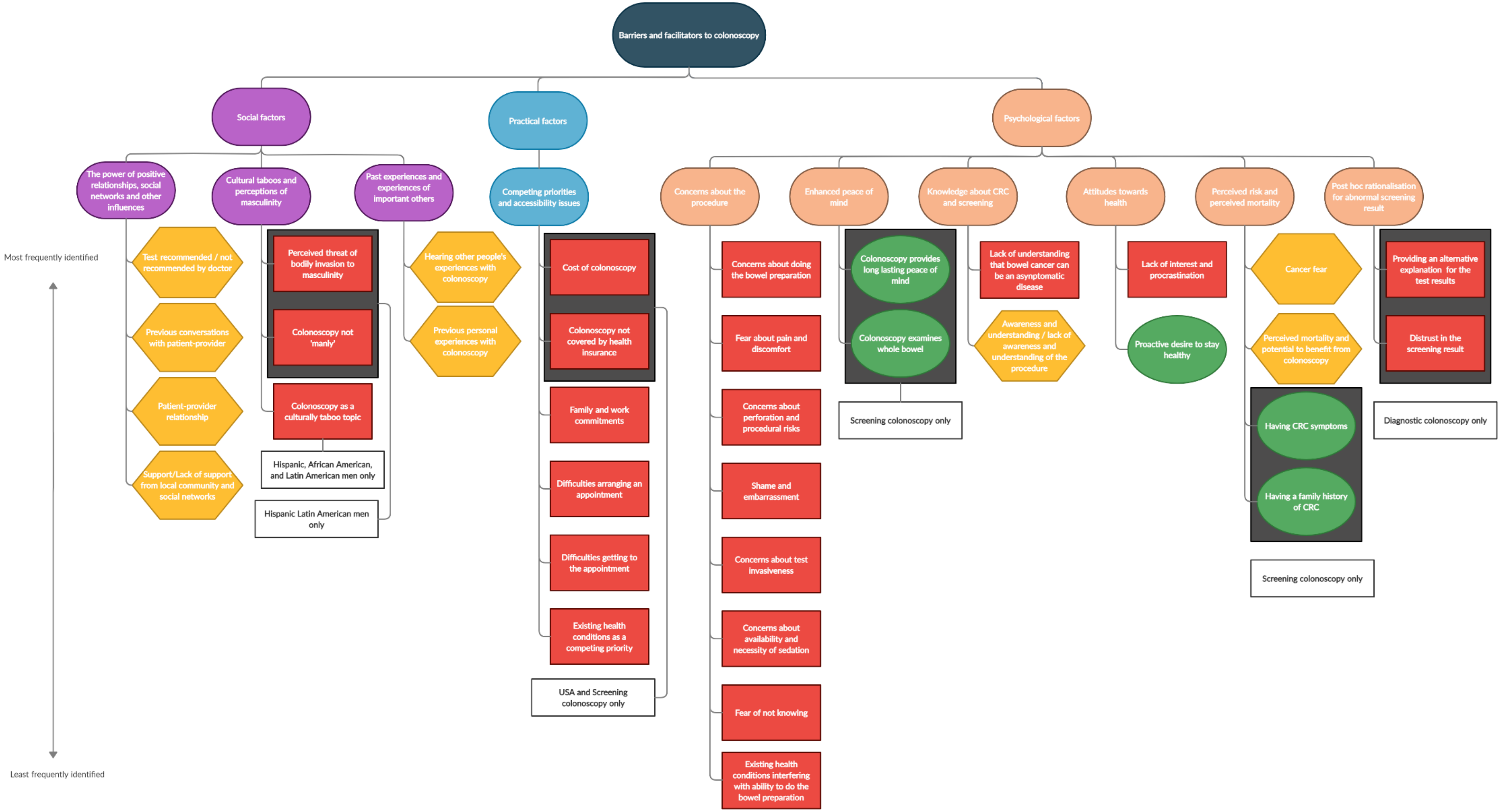

Supplement: Supplementary file 1 — Supplementary material. [file mmc1.pdf]
