# Supplementary figures and images for: Barriers and facilitators to colonoscopy following fecal immunochemical test screening for colorectal cancer: A key informant interview study
Source: Patient Educ Couns. 2022 Jun;105(6):1652–62. doi: 10.1016/j.pec.2021.09.022 (PMC9214549; doi:10.1016/j.pec.2021.09.022)

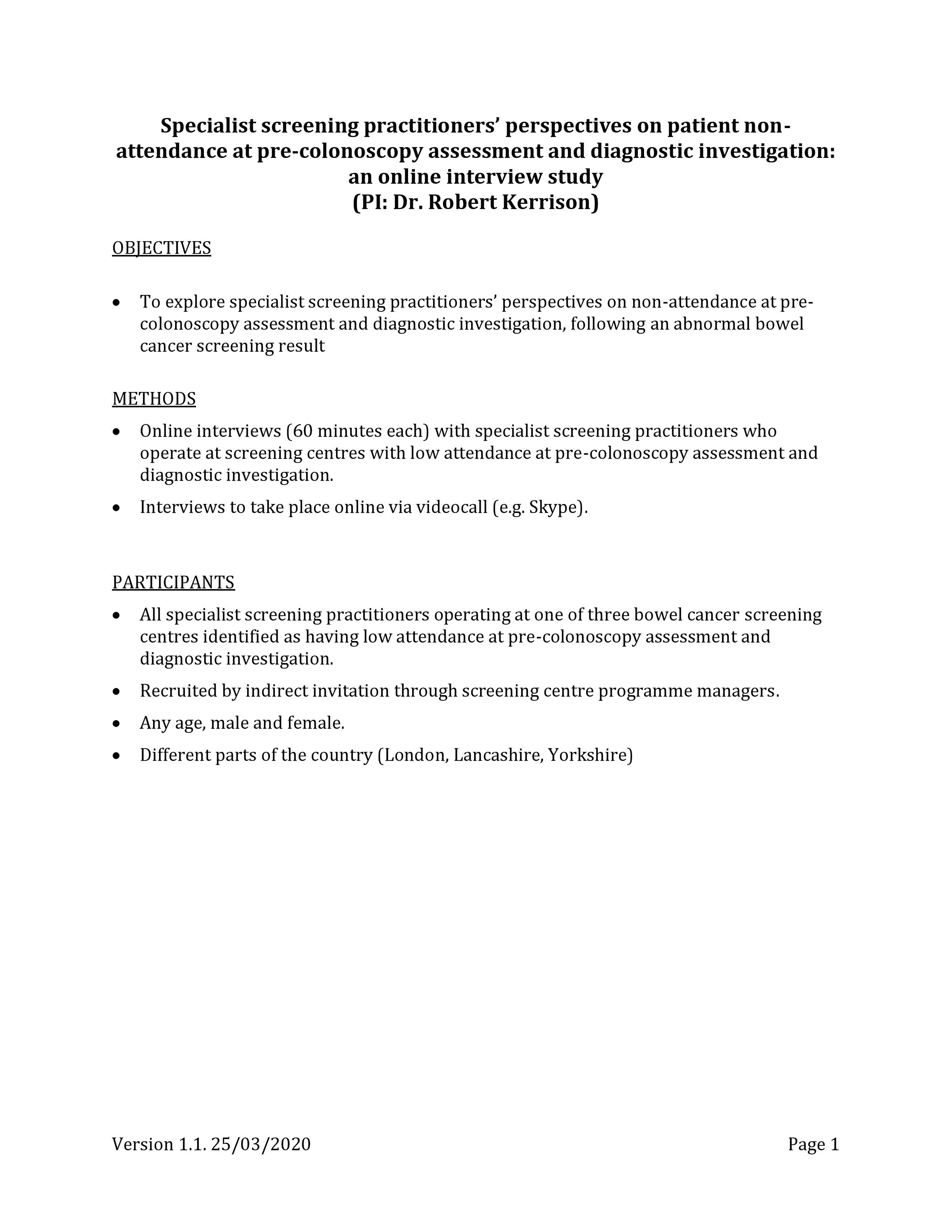

Supplement: Supplementary Fig S1 [file mmc3.jpg]

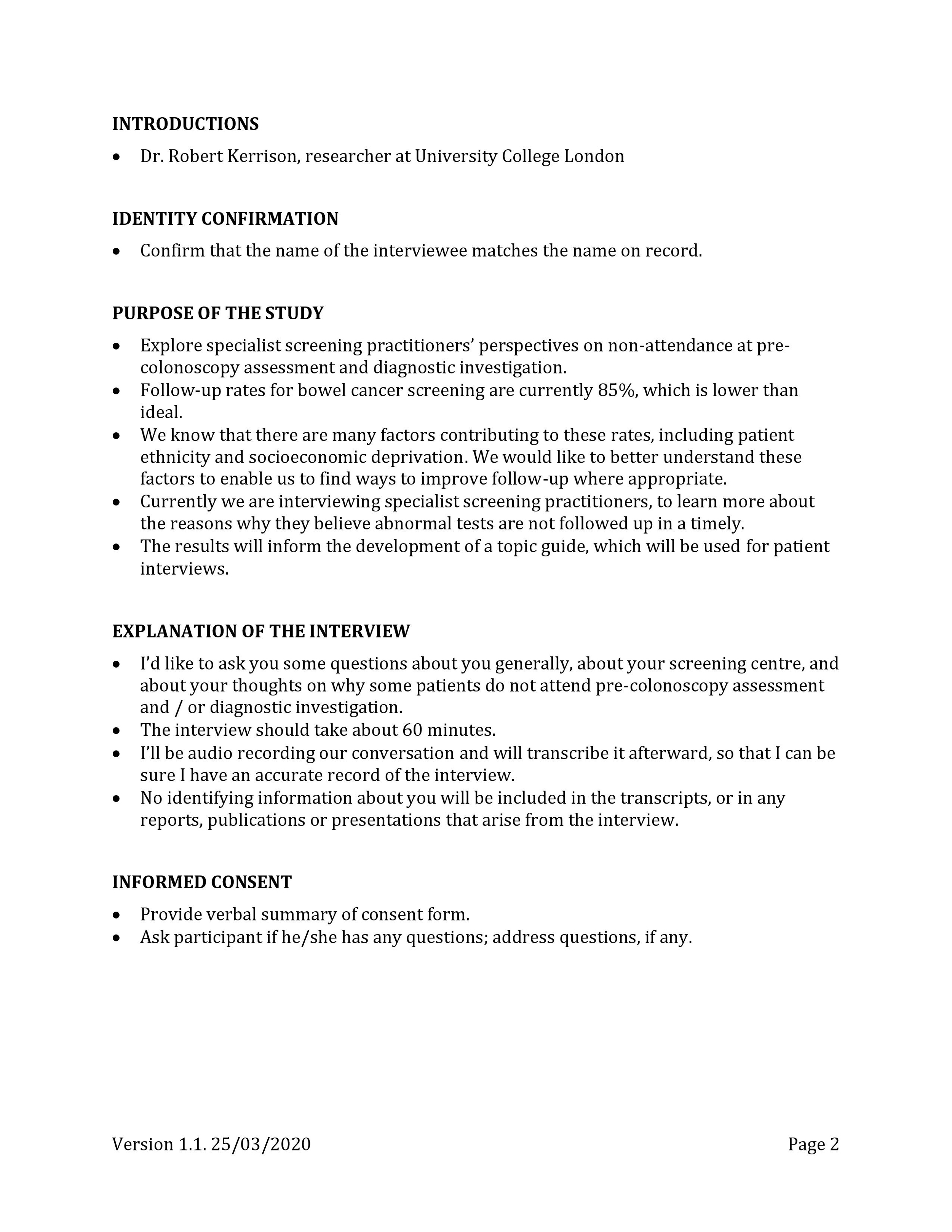

Supplement: Supplementary Fig S2 [file mmc4.jpg]

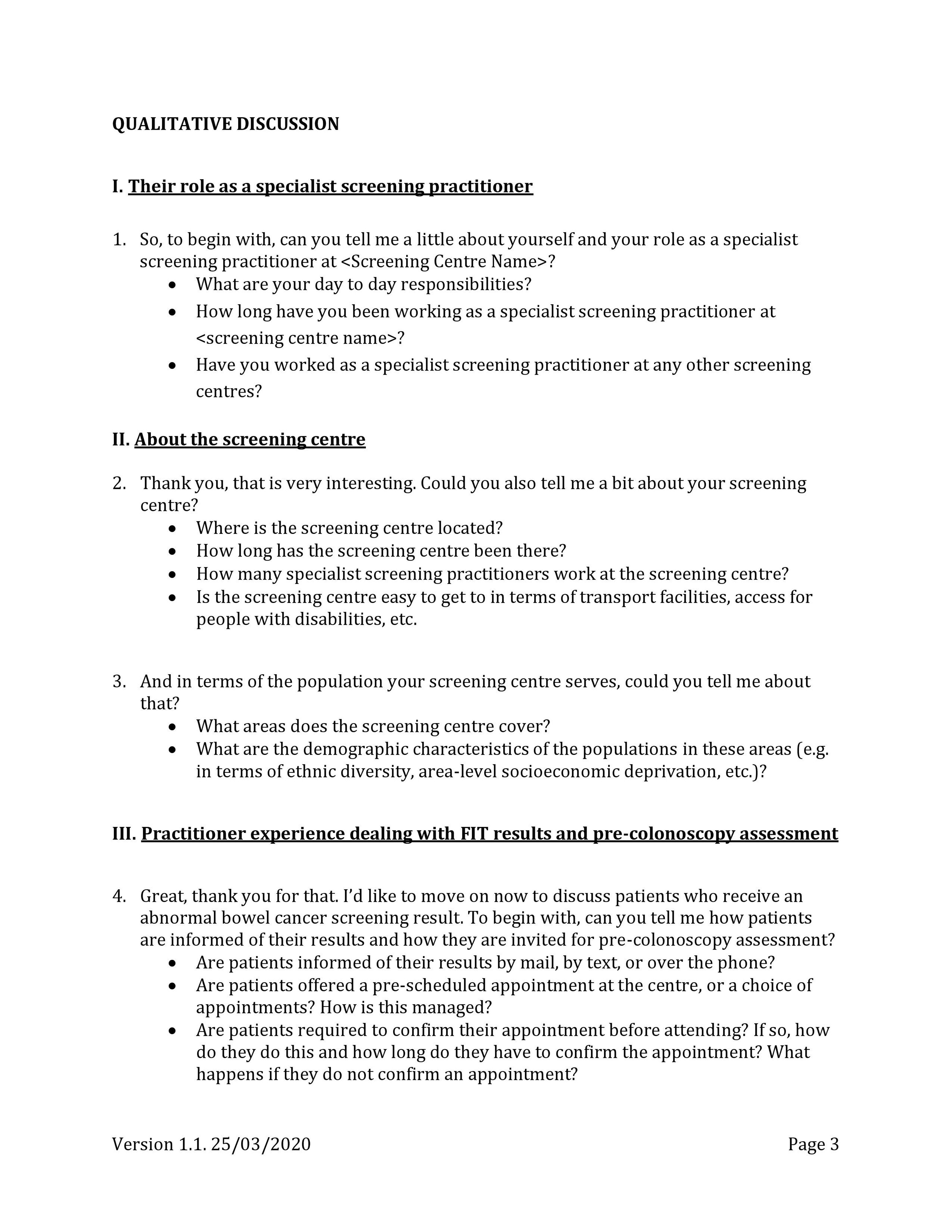

Supplement: Supplementary Fig S3 [file mmc5.jpg]

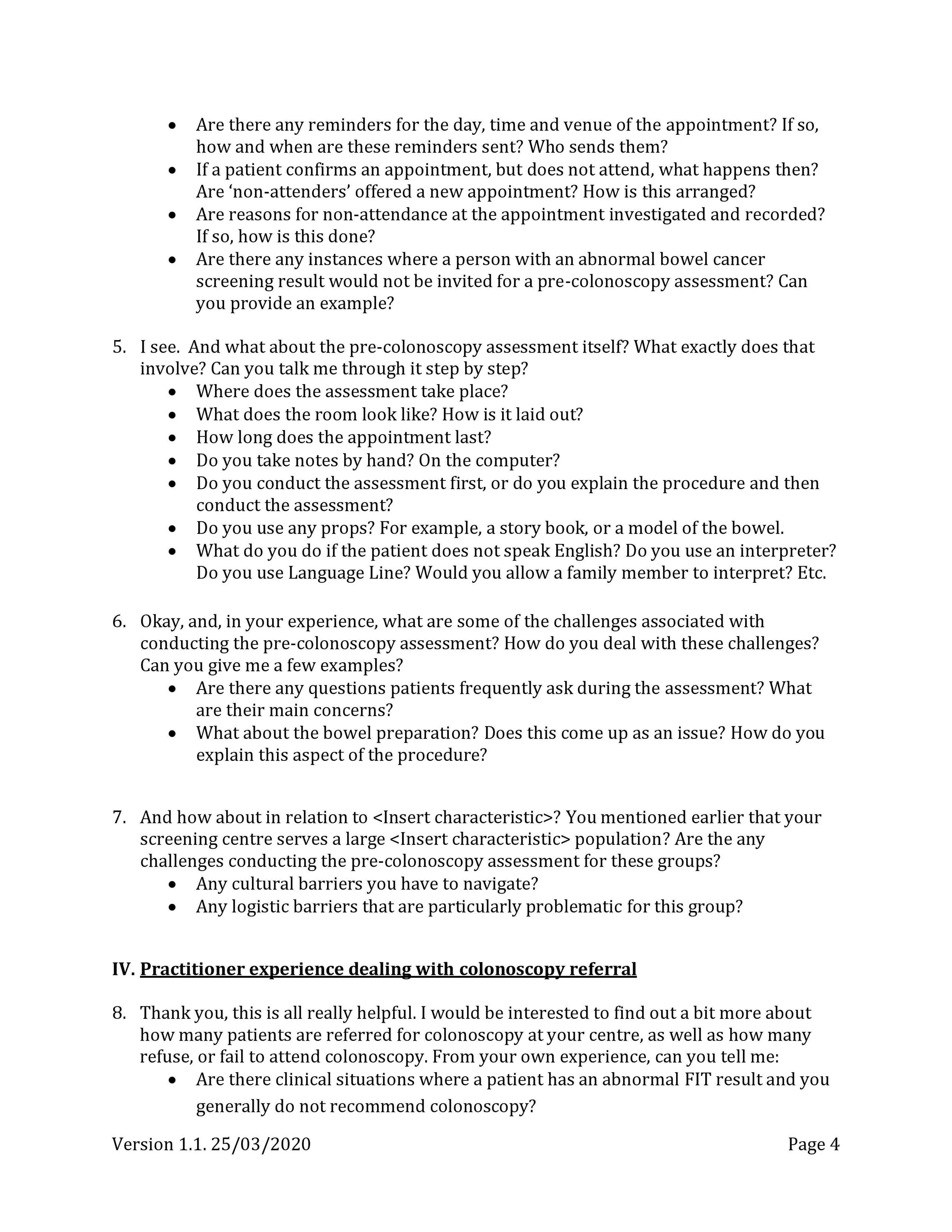

Supplement: Supplementary Fig S4 [file mmc6.jpg]

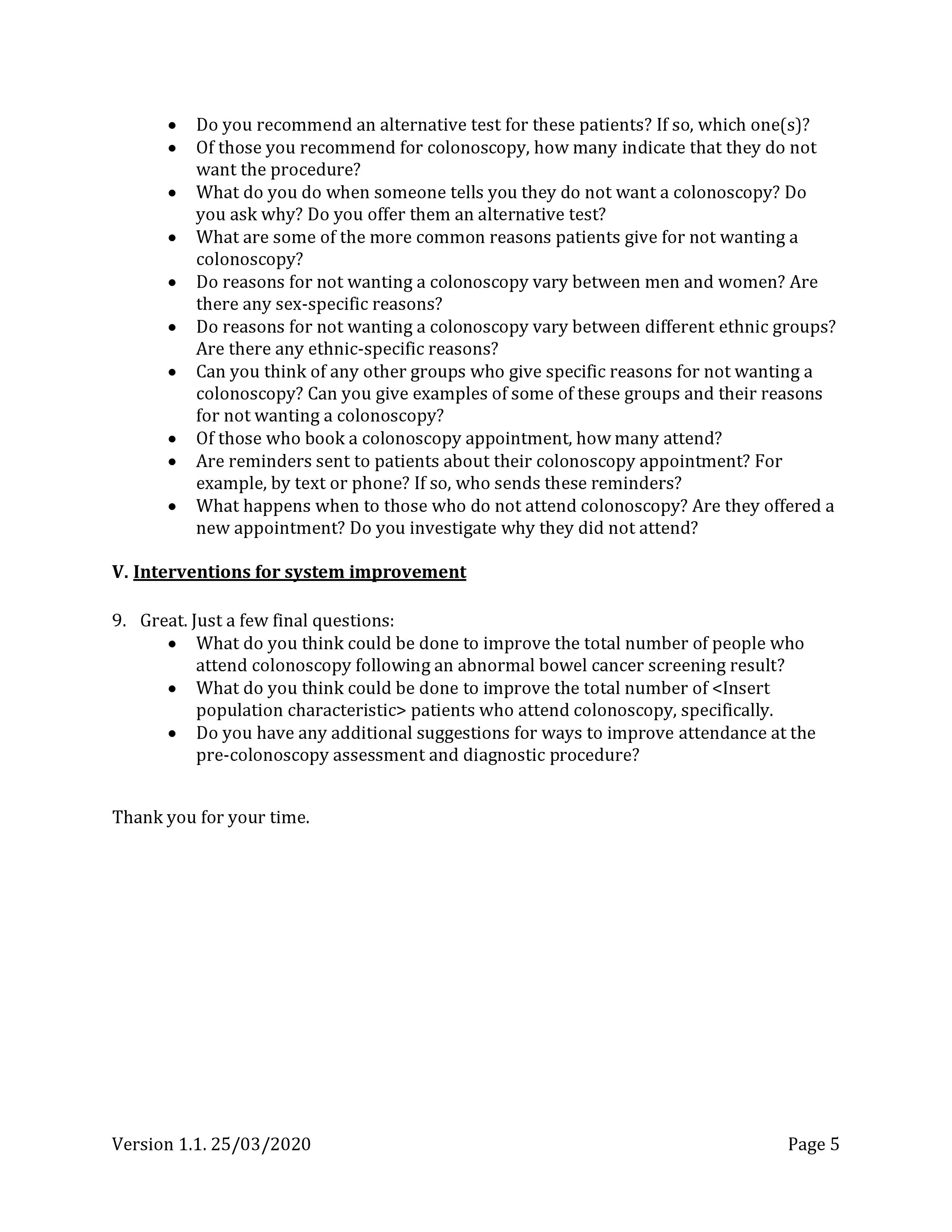

Supplement: Supplementary Fig S5 [file mmc7.jpg]
